# PW01-011 – Exertional leg pain and spondyloarthropathy on FMF

**DOI:** 10.1186/1546-0096-11-S1-A64

**Published:** 2013-11-08

**Authors:** Y Rosman, I Eshed, A Livneh, M Lidar

**Affiliations:** 1 Sheba Medical Center, Ramat Gan, Israel

## Introduction

Exertional leg pain (ELP) is a characteristic musculoskeletal manifestation of FMF which forms one of the minor criteria for its diagnosis. Despite being highly prevalent, the pathogenesis of this unique phenomenon has yet to be elucidated. In a previous pilot study we have described MRI changes compatible with enthesopathy in the feet and ankles of FMF patients with ELP.

## Objectives

The primary objective of the study was to define the frequency and characteristics of exertional leg pain in a large cohort of FMF patients. Secondary objectives were to evaluate for additional signs and symptoms of spondyloarthropathy in this patient population and to assess for the overall frequency of spondyloarthropathy in FMF.

## Methods

All consecutive, consenting patients, aged 18-55, arriving at the FMF outpatient clinic and fulfilling the Tel Hashomer criteria for FMF, were included in the study. Patient allocation into study or control groups was based on the presence or absence of ELP, respectively. Following a detailed clinical, laboratory and genetic workup, randomly selected patients (in a ratio of 1:3 in the study and 1:8 among the control groups) underwent an ankle MRI and plain films of the sacroiliac joints.

## Results

The prevalence of ELP among the 170 FMF patients included in the study was 58.5%, with equal gender distribution. Patients with ELP suffered from more frequent attacks that manifested at an earlier age, and which, in addition to the hallmark abdominal attacks, was characterized by an excess of pleural, joint and febrile bouts.

M694V homozygosity was more prevalent among the study group whereas HLAB27 carriage was a rarity in both groups. The presence of sacroiliitis on plain radiographs was evident in 40% of the study group patients compared to 14% of the controls, respectively, p=0.01. Furthermore, sacroiliitis grade 3-4 was noted in excess among the study group (16.6% vs. 2.5%, respectively). ELP and male gender were independently associated with sacroiliitis on multivariate logistic regression analysis (OR 11.8, 95% CI 1.65-85.44, p=0.014 and OR 5.49, 95% CI 1.14-26.28, p=0.03, respectively). Signs compatible with enthesopathy on MRI of the ankle were observed in 73.5% of the study and 33.3% of the control group, p=0.04. Enthesopathy, characterized by 2 or more MR characteristic pathological findings was recorded in 55% of the study group and none of the controls. Multivariate logistic regression showed that ELP (OR 13.5, 95% CI 1.97-92.44, p=0.008) and male gender (OR 5.5 95%CI 1.21-24.98, p=0.027) were independently associated with signs of enthesopathy on MRI.

## Conclusion

ELP was found to be one of the most common manifestations of FMF, second in frequency to peritonitis and on par with joint attacks. On top of being a marker for a more severe disease phenotype, ELP was found to be associated with local signs of enthesopathy as well as a higher frequency of sacroiliitis. As such, ELP may be regarded as a manifestation of spondyloarthropathy in patients with FMF.

## Disclosure of interest

None declared

